# Supplementary material for: Complexome profiling of the Chlamydomonas psb28 mutant reveals TEF5 as an early PSII assembly factor
Source: Plant Cell. 2025 Mar 17;37(6):koaf055. doi: 10.1093/plcell/koaf055 (PMC12164590; doi:10.1093/plcell/koaf055)
Supplement: koaf055_Supplementary_Data [file koaf055_supplementary_data.zip › psb28_interactive.zip!/plots/intensity/qualityPlots/peptides/RAN1_Cre03.g191050.t1.2.html]

### Cre03.g191050.t1.2

Trivial name: RAN1  
SeqCoverage [median]: 9.00  
Mass: 25.44 kDa  
  
 

| Sequence | MissedCleavage | Mass [Da] | wtIntensity | muIntensity | Score |
| --- | --- | --- | --- | --- | --- |
| HITGEFEKK | 1 | 1087.57 | 5109.98 | 0.00 | 118.67 |
|  |  |  | 5110.0 | 0.0 |  |
